# Nanopore long-read sequencing facilitates accurate diagnosis of *KMT2B*-related dystonia

**DOI:** 10.1186/s13148-026-02243-5

**Published:** 2026-09-17

**Authors:** Ugo Sorrentino, Nazanin Mirza-Schreiber, Martin Pavlov, Fatemeh Peymani, Petra Havrankova, Elisabetta Indelicato, Tereza Serranová, Lukas Kunc, Sandy Lösecke, Erik Tilch, Sebastian Eck, Holger Prokisch, Juliane Winkelmann, Sylvia Boesch, Robert Jech, Elisabeth Graf, Konrad Oexle, Michael Zech

**Affiliations:** 1 Institute of Human Genetics, Technical University of Munich, School of Medicine and Health, Munich, Germany; 2 Institute of Neurogenomics, Helmholtz Zentrum MünchenDeutsches Forschungszentrum Für Gesundheit Und Umwelt (GmbH), Ingolstädter Landstraße 1, Neuherberg, 85764 Munich, Germany; 3 Neurogenetic Systems Analysis Group, Institute of Neurogenomics, Helmholtz Munich, Neuherberg, Germany; 4 Department of Neurology, Charles University, 1st Faculty of Medicine and General University Hospital in Prague, Prague, Czech Republic; 5 Department of Neurology, Center for Rare Movement Disorders Innsbruck, Medical University of Innsbruck, Innsbruk, Austria; 6 Deutsches Zentrum Für Kinder- und Jugendgesundheit, Munich, Germany; 7 Deutsches Zentrum Für Psychische Gesundheit, Munich, Germany; 8 Munich Cluster for Systems Neurology, SyNergy, Munich, Germany; 9 Institute for Advanced Study, Technical University of Munich, Garching, Germany

**Keywords:** *KMT2B*, Long-read sequencing, Nanopore, Episignature, Challenging variation, Streamlined diagnostics

## Abstract

Diagnosis of *KMT2B*-related dystonia remains challenging due to the high prevalence of variants of uncertain significance and technological constraint of short-read pipelines. To overcome these limitations, we integrated nanopore-based long-read sequencing with a validated *KMT2B*-episignature classifier to perform simultaneous genetic and epigenetic profiling for three cases with prior uncertain *KMT2B*-related findings. Our workflow detected characteristic deviations in the *KMT2B*-episignature score in two cases with previously unresolved or missed *KMT2B* variants, while refuting an ambiguous indel call. Combining genomic-variant detection with DNA-methylation analysis eliminated the need for sequential testing and enhanced accurate diagnosis of *KMT2B*-related dystonia, offering a basis for streamlined epigenetics-guided diagnostics.

**Supplementary Information:**

The online version contains supplementary material available at https://doi.org/10.1186/s13148-026-02243-5.

## Introduction

*KMT2B*-related dystonia is a major cause of early-onset generalized dystonia [1, 2]; however, achieving a definitive molecular diagnosis can remain challenging. The diagnostic gap often results from the limitations of current sequencing techniques [3]. The genomic architecture of *KMT2B*—37 exons spanning 20,876 bp, comprising several GC-rich regions—renders the locus susceptible to sequencing artefacts and missed calls in standard pipelines [4]. Furthermore, the interpretation of non-truncating alleles poses challenges [5], leaving a high proportion of variants of uncertain significance (VUSs) [6] that impede clinically relevant interpretation. Such diagnostic doubts may put substantial burden on affected families, causing prolonged uncertainty and, crucially, delaying access to precision clinical management. Given that *KMT2B*-related disorders show a favourable response to deep brain stimulation (DBS) and frequently involve comorbidities requiring tailored interventions [5], rapid and accurate diagnosis is important.

The known function of the ubiquitously expressed KMT2B protein is to transfer methyl groups to the lysine residue located at position 4 of histone H3 (H3K4), ultimately regulating chromatin accessibility and thus, indirectly, methylation of cytosine-phosphate-guanine (CpG) islands in the DNA sequence. Pathogenic genomic variants affecting KMT2B expression and function lead to the establishment of a disease-specific CpG methylation pattern, or ‘episignature’. Episignature analysis has emerged as a powerful tool to prioritize and reclassify disease-associated variants [7, 8], including *KMT2B* variants [9, 10]. However, traditional array-based methylomics are typically performed as secondary, standalone tests. This sequential approach is both time-consuming and resource-intensive, making integrated diagnostics impractical. We have previously demonstrated in a proof-of-concept case that Oxford Nanopore Technologies (ONT)-based long-read sequencing (LRS) can bridge this gap by simultaneously capturing both the causal genetic variant and the associated characteristic epigenetic profile from a single blood sample [11].

In this study, we provide further evidence for this “one-test-fits-all” approach [12, 13]. We present three unrelated patients with unresolved dystonia phenotypes in whom ONT-LRS facilitated accurate diagnostic assessment in the context of (suspected) *KMT2B*-related disorder by integrating high-fidelity single-nucleotide variant (SNV)/indel detection with real-time methylation profiling. Our results underscore the feasibility of ONT-LRS as a comprehensive primary diagnostic tool for dystonia associated with *KMT2B* variants.

## Patients and methods

### Subjects

We included three unrelated individuals (one female and two males) presenting with different forms of dystonia who remained molecularly unresolved following standard-of-care diagnostic procedures [14]. These subjects were enrolled through our research program for rare monogenic dystonias and dystonia-diagnostics development at the Technical University of Munich and Helmholtz Munich (Munich, Germany) [4, 14]. The series was specifically selected to represent diverse diagnostic challenges: case 1 harboured a missense VUS in *KMT2B*, case 2 presented with an ambiguous indel call requiring high-fidelity validation, and case 3 exhibited a clinical phenotype suggestive of a *KMT2B*-related disorder despite negative findings in previous standard pipeline analyses [14]. Prior to enrolment, all patients had undergone whole-exome sequencing (WES) using established protocols for variant analysis based on minor allele frequency and predicted impact on protein [4, 14]. These cases were analysed in 2025 and represent unpublished findings that were not included in our recent whole-cohort reporting [4]. The study was conducted in accordance with the Declaration of Helsinki, and written informed consent was obtained from all participants or their legal guardians under protocols approved by involved institutional ethics review boards.

### ONT-LRS genomic analysis

Genomic DNA was extracted and fragmented as previously reported [11]. Briefly, whole-genome libraries were prepared from 1000 ng of DNA using the ONT Ligation Sequencing Kit V14 and sequenced on a PromethION 24 device (R10.4.1 chemistry) for 72 h. Quality parameters of the analysis of the three index patients are shown in Supplementary Table 1. Basecalling was performed in real time by MinKNOW v26.01.15 (integrated Dorado basecaller) using the super-accurate (SUP) model dna_r10.4.1_e8.2_400bps_sup@v5.2.0, with simultaneous calling of 5-methylcytosine and 5-hydroxymethylcytosine as base modifications recorded in the unaligned BAM files. Reads were partitioned on-instrument into pass and fail bins by mean read quality (Q ≥ 10); only pass reads were used for downstream analysis. For alignment, we used the GRCh38 no-alt analysis set as reference (GenBank assembly GCA_000001405.15). Modified-base-tagged reads were aligned to the reference with minimap2 v2.28-r1209 using the Nanopore long-read preset, preserving base-modification tags; alignments were coordinate-sorted, indexed and summarised with SAMtools v1.22.1. Variant calling (SNVs/indels) was performed using the automated “wf-human-variation” pipeline (v2.8.0) incorporating Clair3 v1.0.8, as previously described [11]. Variants were phased and reads were tagged by haplotype with *longphase* (as bundled in wf-human-variation v2.8.0; –phased). SNVs and indels were functionally annotated with SnpEff and augmented with gnomAD v3.1.2 population allele frequencies using echtvar. SNVs were further annotated with Ensembl VEP, including the SpliceAI, pLI, and LoFtool plugins for variant prioritisation.

#### ONT-LRS DNA methylation extraction and CpG selection

To assess the epigenetic impact of *KMT2B* variants, we aggregated genome-wide ONT DNA methylation information per reference CpG into bedMethyl summaries from aligned BAM files using *modkit* (as bundled in wf-human-variation v2.8.0; --mod module). Because reads were haplotagged, methylation was resolved per haplotype, enabling allele-specific methylation analysis. Beta values were calculated separately for haplotype 1, haplotype 2, and for reads that could not be confidently assigned to either haplotype, and then combined into a single per-site, per-sample beta value by pooling counts across both haplotypes and the ungrouped category. For each CpG site, 5-methylcytosine (5mC) and 5-hydroxymethylcytosine (5hmC) calls were combined into a single “modified calls” count, and the beta value was calculated as the number of modified calls divided by the total number of valid (modified plus unmodified) calls at that position.

We then evaluated the suitability of the 113 CpG sites from our previously defined microarray-derived *KMT2B*-related dystonia episignature [9–11]. The pre-selected CpG sites were subjected to two sequential filtering steps: first, of the 113 CpG sites, 104 could be unambiguously mapped to the ONT-derived call set and retained for downstream filtering, while the remaining 9 sites could not be resolved due to missing CpG identifiers or mapping discrepancies and were therefore removed. Second, each of the remaining 104 CpG sites was retained for downstream analysis only if it achieved a minimum read-depth of 10× in every individual sample; sites not meeting the read-depth threshold in one or more samples were excluded. Applying this combined coverage and read-depth criteria ultimately led to the selection of 80 post-quality control CpG sites.

### SVM classifier re-training, validation, and application

To ensure the robustness of the methylation-based classifier under the reduced, ONT-compatible set of 80 CpG sites, the previously published support vector machine (SVM) classifier (R (version 4.4.1) e1071 package (version 1.7.17)), originally trained on EPIC.v1 array data using all 113 episignature CpG sites [9–11], was retrained using ONT data obtained from the same 38 controls and 9 cases from the original array dataset [9].

The re-trained model was then validated on the previously established EPIC array validation set comprising age- and sex-matched individuals [9], demonstrating preserved performance despite the reduced feature set, with all cases and non-*KMT2B*-related dystonia samples being correctly classified according to their original diagnostic labels [9, 11]. Finally, the retrained classifier was applied to the three current patients to estimate their probabilities of being a *KMT2B*-related dystonia case. Because the retrained classifier required complete data across all 80 CpG sites, and all three ONT samples passed the per-sample coverage and read-depth filters described above, no imputation of missing methylation values was required for classification. Classification was performed in R using the predict() function from the e1071 package, with class-membership probabilities obtained via Platt scaling of the underlying SVM decision values.

## Results

### Clinical characteristics and baseline molecular findings

**Fig. 1:**
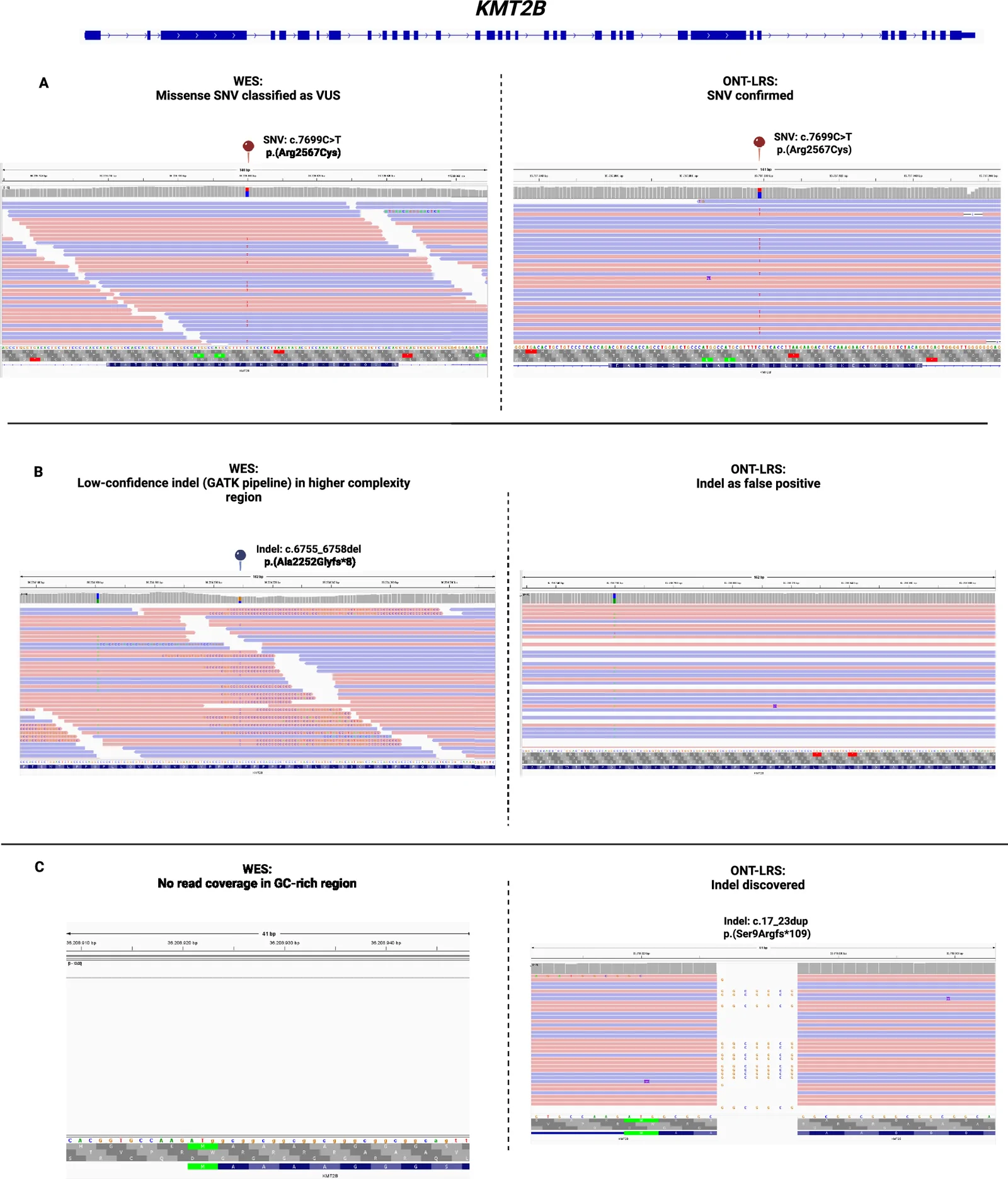
ONT-LRS-based *KMT2B* variant analysis for three cases with dystonia. Visualizations of genomic alignments of WES data and ONT-LRS data in the IGV software are shown for each case. *KMT2B*’s structure is illustrated at the top. WES variant annotations were based on genome assembly GRCh37/hg19, whereas ONT-LRS data were analysed according to GRCh38/hg38. Reads are coloured according to read strand, using the standard IGV colour coding. (**A**) In case 1, the heterozygous missense SNV c.7699C>T, p.(Arg2567Cys) was concordantly identified by both WES and ONT-LRS. (**B**) In case 2, WES flagged a low-confidence heterozygous indel (c.6755_6758del, p.Ala2252Glyfs*8) that was absent upon ONT-LRS, with high-fidelity long-read data showing no evidence of the variant at either allele, consistent with a false-positive WES call. (**C**) In case 3, WES yielded no coverage over the GC-rich region of the beginning of *KMT2B* exon 1, whereas ONT-LRS identified the heterozygous, recurrent [17] pathogenic frameshift indel c.17_23dup, p.(Ser9Argfs*109) in this sequencing “blind spot” [4]. GATK, Genome analysis toolkit; IGV, Integrative genomics viewer; indel, Short insertion/deletion; ONT-LRS, Oxford Nanopore Technologies-based long-read genome sequencing; SNV, Single-nucleotide variant; WES, Whole-exome sequencing

Case 1 was a 49-year-old male who presented with adult-onset dystonia, a phenotype typically associated with idiopathic rather than monogenic aetiologies [14, 15]. Clinical symptoms started at the age of 31 years with an irregular jerky action tremor predominantly affecting the right upper limb, which was initially interpreted as a functional movement disorder. Subsequently, the condition progressed to affect both upper limbs, with later appearance of cervical dystonia at the age of 48, in the absence of major neurodevelopmental impairment or relevant cognitive deficits. Initial singleton WES identified a novel heterozygous *KMT2B* missense variant, NM_014727.3: c.7699C>T, p.(Arg2567Cys) (Fig. 1A), which was absent from gnomAD v4 and predicted to affect an invariant amino-acid residue in the C-terminal part of the protein. Since parental DNA samples were not available, the inheritance pattern could not be established, and the variant was classified as a VUS [6]. Case 2, a 4-year-old male, presented with a complex neurodevelopmental phenotype comprising infantile-onset generalized dystonia, intellectual disability, and developmental delay. While the dystonia remained non-progressive, the clinical course was characterized by emerging ataxic features and intermittent choreoathetosis. Singleton WES initially flagged a potentially relevant but low-confidence *KMT2B* indel, NM_014727.3: c.6755_6758del, p.(Ala2252Glyfs*8) (read-depth at variant position: 29-fold, variant allele fraction: 10%, genotype quality: 40) (Fig. 1B). The technical quality of the call remained ambiguous, complicating its clinical interpretation [3]. Case 3 was a 15-year-old female with a classic *KMT2B*-related presentation, including childhood-onset generalized dystonia starting in the lower extremities, microcephaly, and intellectual disability. The patient showed significant motor improvement following bilateral GPi-DBS at age 11. Despite the suggestive clinical phenotype [5], standard trio-WES analysis failed to identify a causative variant (Fig. 1C).

### ONT-LRS and epigenetic profiling

**Fig. 2:**
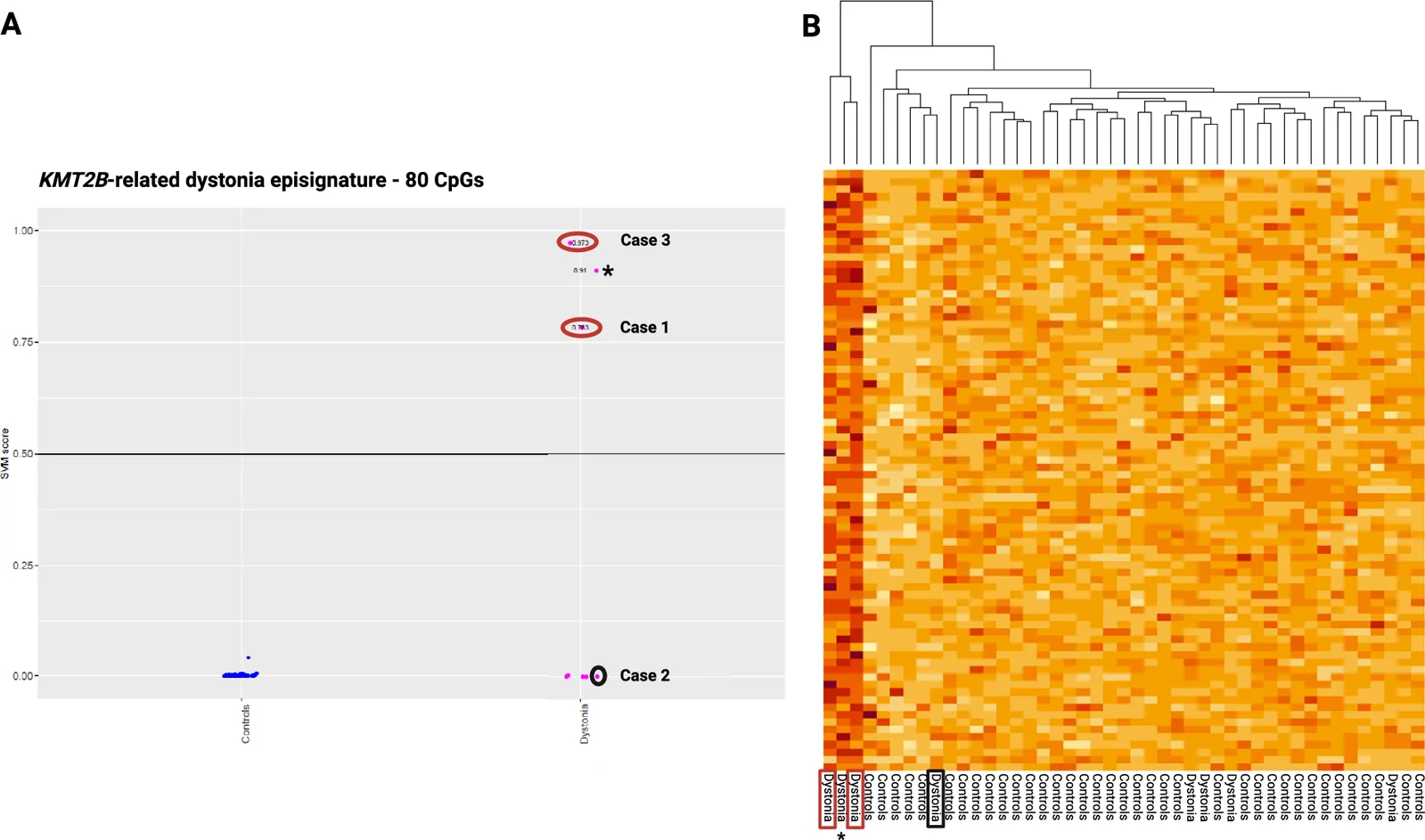
ONT-LRS-based *KMT2B* episignature profiling for three cases with dystonia. Methylation analysis at 80 CpG sites contributing to the core *KMT2B*-related dystonia episignature [11] was performed on ONT-LRS data for cases 1–3, controls, dystonia cases without *KMT2B* rare non-synonymous variants, and one positive control with a causal *KMT2B* variant and a known positive episignature derived from ONT-LRS [11]. For each sample, the classifier score reported in the plot corresponds to the SVM-derived class-membership probability based on the distribution of beta values across selected CpG sites. (**A**) SVM-based episignature scoring for the three here-reported cases compared to 37 control subjects and four dystonia-affected individuals without rare non-synonymous *KMT2B* variants. The previously reported case with positive episignature derived from ONT-LRS [11] is highlighted by an asterisk. The horizontal line at SVM score 0.5 denotes the classification threshold; scores above this threshold indicate a higher likelihood of carrying a disease-related *KMT2B* alteration. Cases 1 (SVM score: 0.783) and 3 (SVM score: 0.973) scored above the threshold, while case 2 clustered strictly among controls, consistent with the absence of a pathogenic *KMT2B* variant. (**B**) Hierarchical clustering based on ONT-LRS-derived methylation levels at the 80 episignature-defining CpG sites, including all three current cases alongside control subjects and dystonia-affected individuals without rare non-synonymous *KMT2B* variants, as well as the positive control (asterisk) [11]. Cases 1 and 3 are highlighted by red boxes at the bottom of the heatmap, while case 2 by a black box. Colour scale represents beta values (dark red, hypermethylation; light yellow, intermediate methylation). Abbreviations: CpG, cytosine-phosphate-guanine dinucleotide; ONT-LRS, Oxford Nanopore Technologies–based long-read sequencing; SVM, support vector machine

In case 1, ONT-LRS confirmed the heterozygous *KMT2B* c.7699C>T, p.(Arg2567Cys) missense variant (local read-depth: 33-fold) (Fig. 1A). Simultaneous DNA-methylation profiling revealed a positive *KMT2B*-episignature readout (SVM probability score: 0.783), supporting the reclassification of the variant as likely pathogenic [6, 9] (Fig. 2A, B). This finding underscored an atypical, milder clinical manifestation of *KMT2B*-related disorder, potentially mediated by hypomorphic effects of specific C-terminal amino acid substitutions [16]. For case 2, automated variant calling (Clair3) of high-fidelity long-read data (local read-depth: 33-fold) indicated the absence of the previously suspected *KMT2B* indel. Visual inspection via the Integrative Genomics Viewer (IGV) confirmed the initial WES finding as a false positive (Fig. 1B). Concordantly, readout of the *KMT2B*- episignature was normal (SVM probability score: 0) (Fig. 2A, B), with the sample clustering strictly among controls; the molecular aetiology for this patient remained currently unresolved. In case 3, ONT-LRS successfully identified heterozygosity for a recurrent [17], pathogenic indel in exon 1 of *KMT2B*, NM_014727.3: c.17_23dup, p.(Ser9Argfs*109) (local read-depth: 35-fold) (Fig. 1C). This region represents a known sequencing “blind spot” for several enrichment kits [4], including the Twist exome utilized in the initial diagnostic screening [18]. The pathogenicity of this variant was further corroborated by the measurement of an SVM probability score close to the maximal value (0.973), reflecting an abnormal *KMT2B* episignature readout from the patient´s sample (Fig. 2A, B).

## Discussion

The molecular diagnosis of *KMT2B*-related dystonia is of significant clinical importance, as it directly informs therapeutic decisions, most notably the initiation of DBS, to which the affected subjects often show an excellent response [2, 5]. However, as demonstrated by our present series, the diagnostic trajectory can be hampered by the limitations of short-read sequencing [3] and the complex genomic architecture of the *KMT2B* locus. In this study, we expand upon and confirm our recent single-case observations [11], providing robust evidence that ONT-LRS may represent a “one-test-fits-all” approach [12, 13] for this disorder. A challenge in diagnostics of *KMT2B*-related dystonia is the high prevalence of VUSs [5], particularly novel missense substitutions. In case 1, the identification of a C-terminal missense variant in an adult-onset patient posed an interpretative hurdle [6]. Traditional pipelines often fail to distinguish between rare benign variation and hypomorphic alleles that may cause milder, late-onset phenotypes [3]. By including ONT-LRS-derived methylation information [11], we were able to demonstrate the useful integration of *KMT2B* episignature readouts into ONT-LRS analysis, providing the functional evidence required for reclassification of the detected missense variant as likely pathogenic [6, 9]. This highlights the value of LRS in expanding the known phenotypic spectrum of *KMT2B*-related disorders beyond the classic early-onset generalized presentations. Furthermore, our results reinforce the superiority of LRS in overcoming the technical shortcomings of standard WES [19, 20]. The large size of *KMT2B* and the presence of GC-rich regions, particularly in exon 1, create certain “blind spots” for commercial enrichment kits [4]. Case 3 illustrated this vulnerability: a recurrent pathogenic indel [17] was missed by initial WES but readily identified by the superior mapping capabilities of LRS [13]. Conversely, case 2 demonstrated how LRS can rapidly resolve diagnostic uncertainty by identifying false-positive indel calls that frequently arise in short-read data due to alignment artefacts [3]. The ability of LRS to provide comprehensive indel assessment alongside SNV detection reduces the risk of both false-negative and false-positive reports [19–21]. Perhaps most significantly, this study reinforces the feasibility of simultaneous genetic and epigenetic testing [11, 21–23]. Currently, episignature profiling typically requires secondary, array-based testing, which increases costs and diagnostic turnaround time [21]. We show that ONT-LRS can extract high-resolution methylation data from the same sequencing run used for variant calling. This consolidation into a single workflow streamlines the diagnostic process, making “one-test-fits-all” a practical reality [12, 13].

In conclusion, our data suggest that ONT-LRS should be considered as a primary diagnostic tool for suspected *KMT2B*-related dystonia, particularly when standard pipelines are inconclusive. By resolving VUS, capturing previously inaccessible variants, and providing real-time epigenetic confirmation, LRS offers a comprehensive solution that can shorten the diagnostic odyssey for affected families and facilitates timely, precision clinical management.

## Supplementary Information

Additional file1 (DOCX 13 kb)

## Data Availability

All data relevant to the study are included in the article or in the publications cited. Further details are available upon reasonable request. The Support Vector Machine (SVM) pipeline is undergoing rigorous prospective testing and refinement within an internal larger, independent cohort as part of a dedicated, separate research program. This ongoing validation is essential to ensure the model’s safety, reproducibility, and robustness before general clinical deployment. The research-stage analysis pipeline is available at https://github.com/NazaninMir/Episig_KMT2B_ONT. The version used for the analyses presented in this study is archived at Zenodo (DOI: 22642883, https://zenodo.org/records/22642883).

## Abbreviations

VUS: Variant of uncertain significance
DBS: Deep brain stimulation
ONT-LRS: Oxford Nanopore Technologies-based long-read sequencing
SNV: Single-nucleotide variant
indel: Short insertion/deletion
WES: Whole-exome sequencing
CpG: Cytosine–phosphate–guanine
SVM: Support vector machine
GATK: Genome analysis toolkit
IGV: Integrative genomics viewer
