# Supplementary material for: Nanopore long-read sequencing facilitates accurate diagnosis of KMT2B-related dystonia
Source: Clin Epigenetics. 2026 Sep 17;18:167. doi: 10.1186/s13148-026-02243-5 (PMC13587463; doi:10.1186/s13148-026-02243-5)
Supplement: Supplementary file 1 [file 13148_2026_2243_MOESM1_ESM.docx]

**Supplementary table 1**

| **Patient ID** | **Median genome-wide read depth** | **Mean genome-wide coverage** | **Median read lenght** | **Sample read N50** |
| --- | --- | --- | --- | --- |
| Patient 1 | 39,325x | 98,30% | 2516 bp | 6370 bp |
| Patient 2 | 40,201x | 97,90% | 7773 bp | 15495 bp |
| Patient 3 | 39,104x | 97.6% | 3810 bp | 9785 bp |
